# Implications of leg length for metabolic health and fitness

**DOI:** 10.1093/emph/eoac023

**Published:** 2022-07-21

**Authors:** Meghan K Shirley, Owen J Arthurs, Kiran K Seunarine, Tim J Cole, Simon Eaton, Jane E Williams, Chris A Clark, Jonathan C K Wells

**Affiliations:** UCL Great Ormond Street Institute of Child Health, 30 Guilford Street, London WC1N 1EH, UK; UCL Great Ormond Street Institute of Child Health, 30 Guilford Street, London WC1N 1EH, UK; Department of Radiology, Great Ormond Street Hospital for Children, Great Ormond Street, London WC1N 3JH, UK; UCL Great Ormond Street Institute of Child Health, 30 Guilford Street, London WC1N 1EH, UK; UCL Great Ormond Street Institute of Child Health, 30 Guilford Street, London WC1N 1EH, UK; UCL Great Ormond Street Institute of Child Health, 30 Guilford Street, London WC1N 1EH, UK; UCL Great Ormond Street Institute of Child Health, 30 Guilford Street, London WC1N 1EH, UK; UCL Great Ormond Street Institute of Child Health, 30 Guilford Street, London WC1N 1EH, UK; UCL Great Ormond Street Institute of Child Health, 30 Guilford Street, London WC1N 1EH, UK

**Keywords:** leg length, lean mass, cardiometabolic risk, developmental origins of health and disease

## Abstract

**Background and objectives:**

Several studies have linked longer legs with favorable adult metabolic health outcomes and greater offspring birth weight. A recent Mendelian randomization study suggested a causal link between height and cardiometabolic risk; however, the underlying reasons remain poorly understood.

**Methodology:**

Using a cross-sectional design, we tested in a convenience sample of 70 healthy young women whether birth weight and tibia length as markers of early-life conditions associated more strongly with metabolically beneficial traits like organ size and skeletal muscle mass (SMM) than a statistically derived height-residual variable indexing later, more canalized growth.

**Results:**

Consistent with the ‘developmental origins of health and disease’ hypothesis, we found relatively strong associations of tibia length—but not birth weight—with adult organ size, brain size, SMM and resting energy expenditure measured by magnetic resonance imaging (MRI), dual-energy X-ray absorptiometry and indirect calorimetry, respectively.

**Conclusions and implications:**

Building on prior work, these results suggest that leg length is a sensitive marker of traits directly impacting metabolic and reproductive health. Alongside findings in the same sample relating tibia length and height-residual to MRI-measured pelvic dimensions, we suggest there may exist a degree of coordination in the development of long bone, lean mass and pelvic traits, possibly centered on early, pre-pubertal growth periods. Such phenotypic coordination has important implications for fitness, serving to benefit both adult health and the health of offspring in subsequent generations.

## INTRODUCTION

Taller stature and longer leg length have been linked with a range of adult cardiometabolic health outcomes, such as lower blood pressure and insulin resistance, and reduced risk of ischemic heart disease and type 2 diabetes [1–4]. Conversely, longer trunk length tends to show relatively weak, or opposite, associations with these outcomes. A recent Mendelian randomization study suggested the association of height with lower cardiometabolic risk is causal [5], but the underlying reasons remain poorly understood. In women, greater height and longer leg length are also associated with higher birth weight of offspring [6–8]. Although longer legs indicate increased absolute muscle and bone mass in the lower limbs, these associations are unlikely to fully account for the health benefits. Rather, leg length probably acts as a marker of other traits that impact metabolic outcomes more directly [9].

Leg length has attracted particular interest in life-course epidemiological research because it appears especially sensitive to exposures acting in early life [10, 11]. This sensitivity can be leveraged to investigate the ‘developmental origins of adult health and disease’ (DOHaD), the focus of a field whose early work demonstrated inverse associations between size at birth and cardiometabolic risk [12]. For example, the thrifty phenotype hypothesis [13] proposed that poor growth *in utero* results in ‘brain sparing’ at the expense of other organs, thereby increasing susceptibility to non-communicable disease in later life. Indeed, individuals of both high and low birth weight appear susceptible to later obesity, while low birth weight may predispose to increased visceral fat [14]. However, birth weight is a challenging proxy for fetal growth: it is confounded by variability in neonatal fat mass (FM) and indexes not only growth patterns *in utero*, but also post-natal weight gain, as small babies tend to experience catch-up growth in early infancy [15].

Growth of the lower limb provides a more general marker of early growth conditions, being sensitive to nutritional supply and other environmental exposures in each of fetal life, infancy and early childhood [16, 17]. Evidence suggests that tibia length may be particularly sensitive to environmental conditions in these early periods (i.e. relative to the femur or total leg length) [16, 18, 19]. Tibia length also has advantages over total leg length, calculated as the difference of height and sitting height, as sitting height is biased by variability in gluteal fat [18]. Acknowledging this limitation, the ratio of leg length to total height (relative leg length) appears to be independent of size at birth; hence, it may act as a valuable marker specifically of variability in post-natal growth [9, 17]. Understanding associations of different components of height with physical markers of metabolism and homeostasis could shed further light on the DOHaD hypothesis.

We addressed this by exploring more comprehensively associations of birth weight and components of adult height with adult body composition outcomes including skeletal muscle mass (SMM), FM and organ size, all of which may influence cardiometabolic risk. We incorporated three main growth markers in our analysis: birth weight, tibia length and height residual, the latter being a derived measure of height variability that is statistically independent of variability in tibia length. This approach considers the following: that if there is a component of growth strongly affected by experience in early life, for which the tibia is a good marker, there may be another axis of variability in growth that reflects later periods, and perhaps to a greater extent than the tibia, genetic factors. Height residual was therefore taken to index the period of development during which growth becomes increasingly canalized (i.e. less susceptible to environmental conditions), potentially beginning as early as 2 or 3 years of age [20, 21] and extending until linear growth cessation.

We analyzed anthropometric and high-quality body composition data in a sample of nulliparous South Asian women aged 20–28 years who were recruited in London, UK, for a larger study on body composition, pelvic phenotype and resting energy expenditure (REE), as reported previously [22, 23]. All participants were healthy, though some South Asian populations have experienced poor growth associated with both elevated cardiometabolic risk and a relatively high frequency of low-birth weight offspring.

The ‘capacity–load’ model [24] assumes that cardiometabolic risk is shaped by two generic traits: ‘metabolic capacity’ contributes to the maintenance of homeostasis, while ‘metabolic load’ challenges homeostatic capacity. Lower capacity and higher load are each expected to increase cardiometabolic risk, as supported by several cohort studies [4, 25]. Moreover, metabolic capacity in women is beneficial for promoting healthy fetal growth. Considering the literature linking (i) birth weight and leg length with later metabolic risk and (ii) maternal leg length with offspring birth weight, we hypothesized that markers of early growth in our sample—that is, adult tibia length and birth weight—would demonstrate stronger associations than height residual with ‘capacity traits’ like skeletal muscle and organ size, but not with ‘load traits’ such as FM.

## METHODOLOGY

Participants were recruited based on the following criteria: they were female, healthy, of South Asian ancestry (Indian, Pakistani, Bangladeshi and Sri Lankan), nulliparous and aged 20–28 years, with a body mass index (BMI) in the range 17–28 kg/m^2^. Subjects self-identified their ancestry and confirmed that their maternal and paternal grandparents were South Asian. Being born outside of South Asia was not an exclusion criterion.

Nulliparous condition avoided confounding by differential parity, while the age range was chosen to avoid phenotypic variability associated with pubertal growth and aging. The BMI criterion sought to focus on those within the body weight range designated as ‘normal’ by excluding women classified as very underweight, very overweight or obese. We shifted our BMI range to the left of the WHO’s typical classification because, in general, Asian populations demonstrate lower mean/median BMI compared with non-Asian populations [26]. Exclusion criteria were health conditions potentially impacting growth, smoking, contraindications for MRI and preterm birth (<37 weeks’ gestation), with the latter attempting to control for the possibility that variability in body composition and/or metabolism outcomes developed in association with preterm birth.

We recruited a convenience sample using posters, online advertisements and word-of-mouth. Data collection took place from March 2015 to May 2016 at UCL Great Ormond Street Institute of Child Health and Great Ormond Street Hospital for Children NHS Trust. All participants attended one to two appointments to complete measurements. The study received ethical approval from a Research Ethics Committee of the NHS Health Research Authority. All participants gave written, informed consent.

Height was measured in duplicate to the nearest 0.1 cm using a wall-mounted stadiometer (Holtain, Dyfed, UK). Weight was measured in duplicate to the nearest 0.01 kg using a scale integral to the BodPod air-displacement plethysmography system (Cosmed, Rome, Italy). Large sliding calipers (Holtain, Crymych, UK) were used to measure in duplicate, to the nearest millimeter, tibia length as the distance from the medial tibial plateau to the inferior edge of the medial malleolus on the left leg only, following standard protocols [19]. We obtained information on birth weight and gestational age by subject recall (i.e. based on subjects’ parents’ recall or birth records held by the family).

Fat-free mass (FFM; here used synonymously with ‘lean mass’) and FM were derived using the four-component model of body composition assessment, as described previously [27]. Dual-energy X-ray absorptiometry (DXA; Lunar Prodigy, GE Medical Systems, Madison, WI, USA) was used to quantify SMM. Subjects underwent a scan of approximately 5 min duration, with the mass of lean, non-fat tissue in the arms and legs provided directly by the DXA system software (Encore, v14.10.022). The basis for using these data as a measure of whole-body SMM, and further details of the method, have been described previously [28].

A Deltatrac II indirect calorimeter (Datex-Engstrom Corp, Helsinki, Finland) was used to measure subjects’ whole-body REE in a post-absorptive state, while in a thermoneutral environment (REE closely correlates with FFM). Following gas calibration of the metabolic cart, O_2_ consumption and CO_2_ production were assessed continuously for 25 min as subjects rested, supine, on a hospital cot under a ventilated plastic canopy. Using these data, REE was calculated in kcal/24 hr units using the equation of Weir [29]: (3.941 × VO_2_) + (1.106 × VCO_2_). Subjects were not measured at a specified point in their menstrual cycle. However, day-of-cycle at data collection (estimated using subject-reported information on recent menstruation and general cycle length) was found not to be associated with measured REE and thus rejected as a potential confounder.

High-resolution 3D imaging of the brain, chest and abdomen was undertaken using a 3-T Siemens Magnetom Prisma scanner (Siemens, Erlangen, Germany). The following were acquired: a T1-weighted MPRAGE sequence for brain volume (TR = 2000 ms, TE = 2.74 ms, flip angle = 8°, voxel size = 1.0 × 1.0 × 1.0 mm isotropic, slices = 240, duration = 5 min); a T2-weighted, turbo spin echo SPACE sequence for the abdomen (TR = 2000 ms, TE = 220 ms, flip angle = variable, voxel size = 1.5 × 1.5 × 1.5 mm isotropic, slices = 144, duration = 7 min) and for the chest, a T2-weighted TrueFISP sequence with breath-hold (TR = 475 ms, TE = 1.53 ms, flip angle = 47°, voxel size = 1.5 × 1.5 × 4.0 mm, gap = 0, slices = 42, duration = 20 s).

T1-weighted MR images were processed and segmented with FreeSurfer (v5.3) to derive brain volume, as described in detail elsewhere [30]. Heart, kidneys, liver and spleen were manually segmented from raw MRI data using the open-source OsiriX DICOM viewer (v8.5). Regions of interest were drawn around the organs of interest in contiguous image slices in each subject dataset. The software automatically calculated organ volume by summing the voxels in the regions of interest and multiplying by the slice thickness. Duplicate organ volumes were derived on different days and averaged. The technical error of measurement for the duplicate measures was 1.9% for the heart, 1.1% for the left kidney, 0.7% for the right kidney and 0.7% for the liver.

### Statistical analysis

Because they scale allometrically with one another, anthropometric and body composition variables were natural log-transformed for analysis. Standardized residuals of the regression of (height—tibia length) on tibia length were used as a ‘height-residual’ variable, taken as an index of growth variability that is statistically independent of tibia length (i.e. representing variability in aspects of growth that are not sensitive—or less sensitive—to early-life influences).

Pearson correlations were calculated for body composition variables and REE with height, height-residual, tibia length and birth weight. Birth weight and tibia length variables were then converted to standard deviation score (SDS) to better compare the magnitude of the three growth markers’ respective associations with organs, tissues and REE.

Data were plotted to visualize associations of body composition variables and REE with tibia SDS and standardized height-residual. A set of multivariable regression models was fitted with a body composition variable (i.e. FFM, FM, SMM, organs and brain), or REE, as the dependent variable. In each model, standardized height residual, tibia SDS and birth weight SDS were included as explanatory variables. The R package relaimpo [31] (v2.2-3) was used to assess the explanatory variables’ relative contribution to *R*^2^ in each model, or in other words, the ‘relative importance’ of each individual regressor for explaining variability in the outcome. Statistical analyses were conducted using the R language and environment for statistical computing (v3.6.1) in RStudio (v1.1.463). Two-tailed tests were considered significant at *P* < 0.05.

## RESULTS

A majority of the 70 female participants recruited were university students living in or around London, UK. Breakdown by ethnicity was 51% Indian, 11% Pakistani, 11% Bangladeshi and 11% Sri Lankan. A further 13% reported mixed ancestry among the four represented countries, while one participant’s ancestors had emigrated to Mauritius from India. Forty-seven percent of the sample were born in South Asia, while most of the others were born in the UK. The measured BMI range was 17–30 kg/m^2^. All but one subject reported a gestational age between 37 and 42 weeks.

With respect to the three main growth markers used, height residual was statistically unrelated to tibia length and correlations of birth weight with height residual (*r* = 0.20, *P* = 0.1) and with tibia length (*r* = 0.003, *P* = 0.9) were weak.

**Figure 1.**
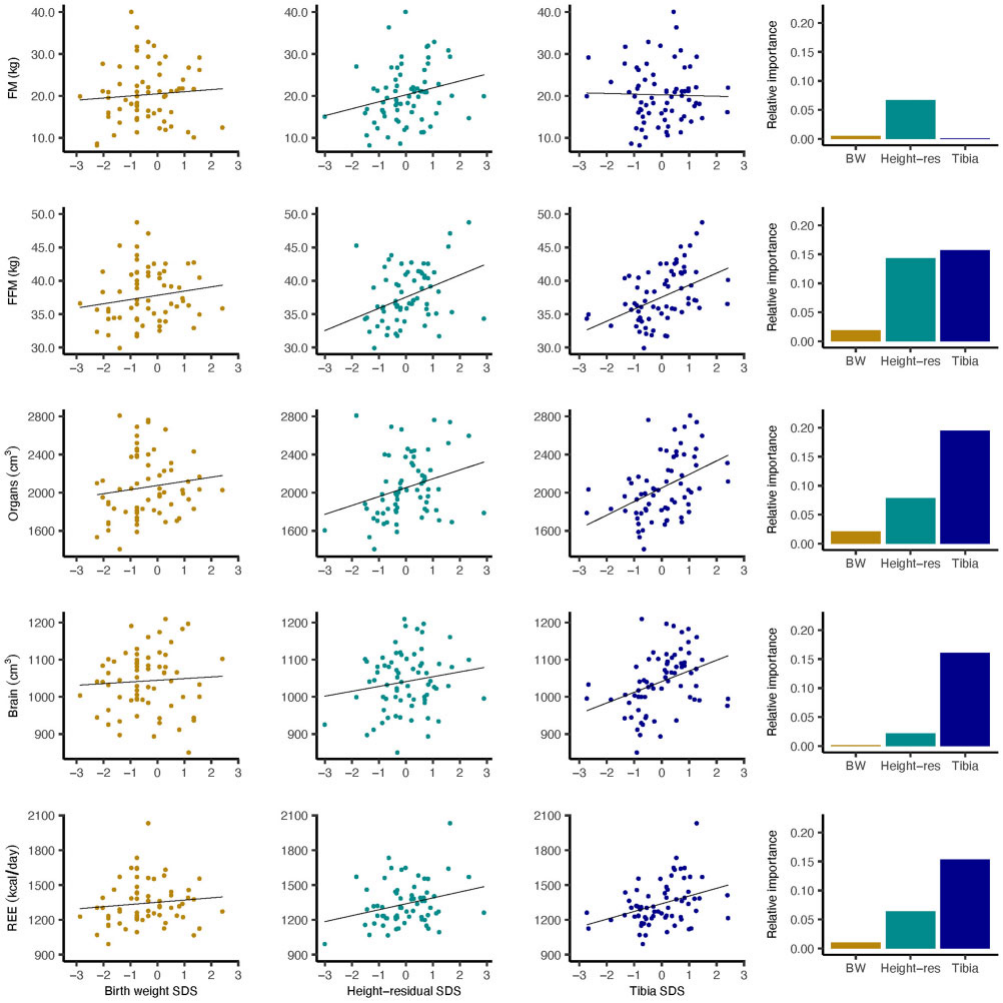
The first three columns from left show plots of unadjusted associations for FM, FFM, organs, brain and REE with birth weight, height residual and tibia, respectively. The far-right column shows bar charts demonstrating the relative contribution to model *R*-squared for tibia, height residual and birth weight in a multiple regression model where that row’s body composition outcome (e.g. FM in the top row) was entered as the dependent variable. The composite ‘organs’ variable is summed heart, liver, kidneys and spleen

**Figure 2.**
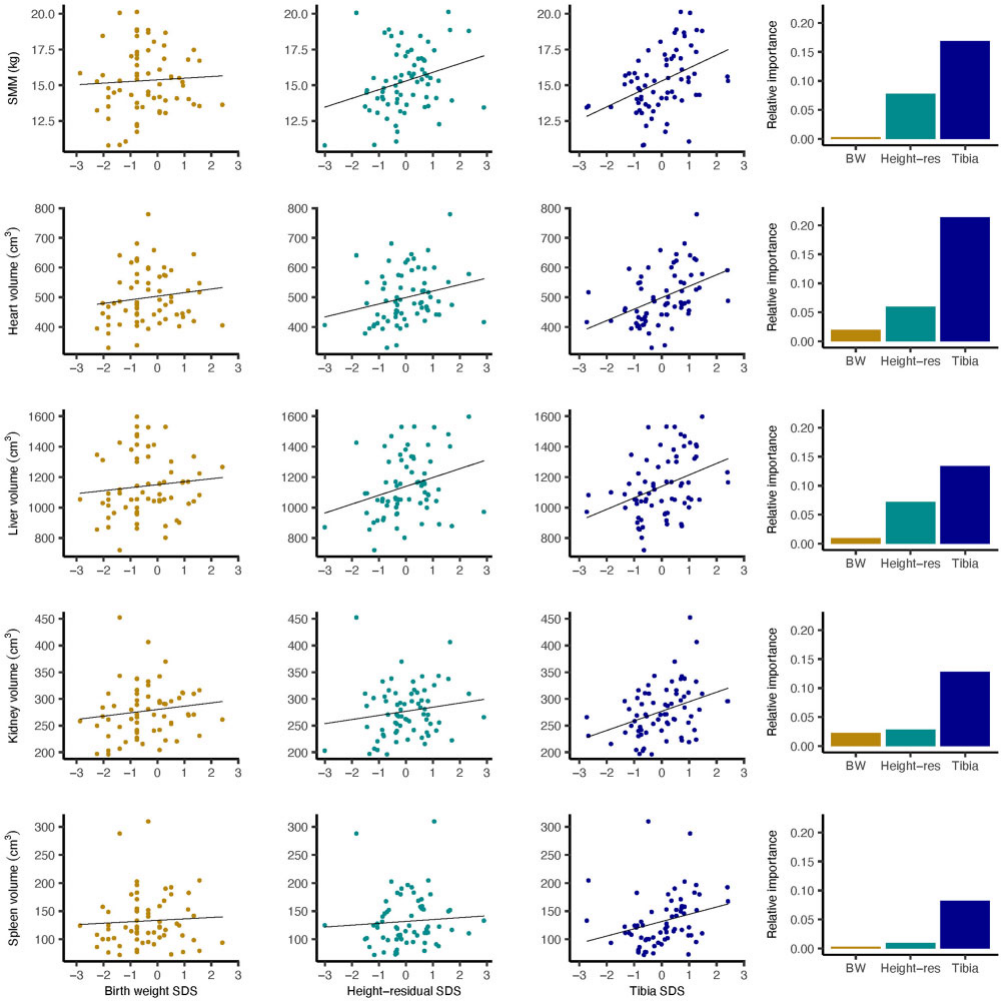
This figure expands on Fig. 1 to visualize relationships between the three growth markers and specific components of FFM. The first three columns from left show plots of unadjusted associations for SMM, heart, liver, kidney and spleen volumes with birth weight, height residual and tibia, respectively. The far-right column shows bar charts demonstrating the relative contribution to model *R*-squared for tibia, height residual and birth weight in a multiple regression model where that row’s body composition outcome (e.g. SMM in the top row) was entered as the dependent variable

Descriptive statistics for the sample and Pearson correlation coefficients for body composition variables, REE, growth markers and total height are given in Supplementary Tables S1 and S2. Plots in Figs 1 and 2 demonstrate that relationships of birth weight with all variables of interest were weak in comparison to those seen for tibia length and height residual. Tibia length was significantly associated with REE and all body composition variables except FM; among the organs, coefficients ranged in size from 0.30 (spleen) to 0.43 (heart). Compared with tibia length, height residual correlated more strongly with FM, with a point estimate of 0.26 and the interval extending to 0.47 at the upper end. Like the tibia, height residual showed significant associations with FFM, SMM, heart volume, liver volume and REE, although coefficients were generally smaller than those seen for the tibia with the same variables.

In Table 1, results of six multiple regression models are given, each with a single body composition variable, or REE, entered independently as the dependent variable. Birth weight, tibia length and height residual, all in SDS, were entered into each model as explanatory variables. Height residual and tibia demonstrated positive associations with several of the same outcomes (i.e. FFM, SMM and organs as a composite variable (summed volumes of the heart, liver, kidneys and spleen)). Estimated beta coefficients (*β*) denote the change in the dependent variable per SD change in the independent variable. In each case, *β* for tibia was comparatively large in size, particularly for SMM and organs, and the *P* values for tibia generally constituted stronger evidence that these data were inconsistent with the null hypothesis of no association [95% confidence interval (CIs) were of similar width].

**Table 1.** Multivariable regression of body composition outcomes and REE on tibia length and birth weight SD scores and standardized height residual^a^

	Independent variables									
	Tibia length SDS			Standardized height residual			Birth weight SDS			
Dependent variable	*β*	*P*	95% CI	*β*	*P*	95% CI	*Β*	*P*	95% CI	Adj. *R*^2^
FM (kg)	0.00	0.97	−0.08, 0.08	0.08	0.05	−0.00, 0.16	0.01	0.87	−0.08, 0.09	0.02
FFM (kg)	0.05	<0.001	0.02, 0.07	0.04	0.001	0.02, 0.06	0.01	0.33	−0.01, 0.04	0.29
SMM (kg)	0.06	<0.001	0.03, 0.09	0.04	0.01	0.01, 0.07	0.00	0.78	−0.03, 0.04	0.21
Organs (cm^3^)	0.07	<0.001	0.04, 0.10	0.04	0.01	0.01, 0.08	0.02	0.31	−0.02, 0.05	0.26
Brain (cm^3^)	0.03	0.002	0.01, 0.05	0.01	0.15	−0.00, 0.03	0.00	0.94	−0.02, 0.02	0.12
REE (kcal/day)	0.05	0.002	0.02, 0.08	0.04	0.02	0.01, 0.07	0.01	0.38	−0.02, 0.04	0.18

a Each row is a regression model with one dependent variable and three independent variables; ‘standardized height-residual’ variable was derived from the regression of (height—tibia length) on tibia length; ‘organs’ variable is summed heart, liver, kidneys and spleen.

In contrast, controlling for tibia length and height residual, associations of birth weight with body composition and REE were weak, with all point estimates close to zero. Figures 1 and 2 show bar charts demonstrating the relative contribution to model *R*^2^ for tibia, height residual and birth weight. The tibia made the greatest relative contribution in all but the FM model, while the contribution of height residual varied across models and that of birth weight was overall negligible.

We further tested associations of body composition and REE with relative leg length, which, as noted above, has been evidenced as a marker specifically of post-natal growth (i.e. independent of fetal growth [9, 17]). Pearson correlation coefficients for body composition variables and REE with relative leg length are given in Supplementary Table S3, with accompanying plots given in Supplementary Fig. S1. Associations of relative leg length with tissue/organ outcomes were generally weak.

## DISCUSSION

This study has documented associations of different growth markers with adult organ size, brain size, SMM and REE. For all organs and for muscle mass, the strongest associations were evident with tibia, whereas a contrasting pattern was evident for body fat, most strongly correlated with height residual. Organs and SMM are highly metabolically active and important for metabolic homeostasis [32, 33], thus helping to explain why longer legs have consistently been linked to more favorable adult cardiovascular health outcomes [1–4]. SMM, for example, is a large, insulin-sensitive tissue with a key role in glucose disposal and low SMM has been associated with increased cardiometabolic risk [34]. Recently, a Mendelian randomization study using UK Biobank and European genome-wide association study data suggested that, accounting for FM, increased FFM was causally protective against cardiometabolic disease [35]. While adding centimeters to one’s tibia may not directly confer health benefits, the extra length does appear to reflect important exposures, as others have posited [9].

Height has for some time been recognized to correlate with organ size in both children and adults [36, 37], but associations of organs with specific components of height have not previously been described. Moreover, our key finding is that tibia length as a marker specifically of early growth correlates more strongly with lean tissue outcomes than does height residual, a marker of later, more canalized growth. These results add further support to the notion that growth conditions in fetal life, infancy and early childhood are critical not only for height, but also for brain and lean mass development. Indeed, antecedents of the brain’s ultimate structural and functional capacity can be traced to the first 3 years of life [38], while early growth faltering is associated with lower lean mass in adulthood [39].

Our results, coupled with those that previously identified leg length-cardiovascular disease links, are consistent with the capacity–load model; namely, crucial components of metabolic capacity (organs, SMM) develop early in life and may later aid in mitigating the deleterious effects of metabolic load (i.e. high glycemic load diets and sedentariness) [24]. Evidence in the literature has suggested that birth weight also scales positively with ‘capacity traits’ [24], however, in this sample we found only weak associations between birth weight, adult lean mass variables and REE. Accounting for tibia length and height residual, all the models gave point estimates for birth weight around zero; therefore, both small negative, or small positive, associations of birth weight with the variables of interest were reasonably compatible with the data, given model assumptions. One potential explanation is that birth weight includes both FM and FFM, and high FM variability [40] may serve to obscure an association of birth weight with adult FFM variables. However, it is important to note that tibia length reflects growth in both fetal and post-natal life [17]; hence, our findings do not discount the importance of fetal development, but rather indicate that birth weight was not a useful marker of this experience in the current sample.

Although perhaps less involved in mitigating metabolic risk than internal organs, SMM and REE, the implications of optimal brain development for health and fitness cannot be overstated. The relatively strong association we observed for tibia length versus height residual is consistent with what is known about the importance of early life growth for brain development, even as various aspects of brain phenotype continue to grow and develop across the life course [38]. As environmentally mediated disruptions to brain development during this period are predicted to have long-term negative consequences for cognition, education, productivity and mental health, pregnancy and infancy have been highlighted as critical periods for targeted intervention [38, 41] and our results further support this.

With respect to reproductive outcomes, our study sample included only nulliparous women; therefore, we were not able to directly test potential intergenerational associations of participants’ tibia length with the birth weight of their offspring. Our results, however, arguably aid in explaining previously identified positive associations of parental—in particular, maternal—leg length with offspring birth weight [6–8], and point to important implications for reproductive fitness. Namely, in addition to the positive impact of mother’s postnatal accumulation of lean mass on her own lifetime metabolic capacity, her increased organ size, greater SMM and greater REE are key to funding the growth of her offspring [42]. Indeed, studies in high-, middle- and low-income countries have reported associations between maternal lean mass and offspring birth weight, while equivalent associations with maternal FM have been weaker or absent (reviewed in Wells [42]). Relative FFM—in particular, internal organ size—explains the majority of inter-individual variation in REE [37] and maternal metabolism has been proposed as the central constraint on fetal growth [42–44].

**Figure 3.**
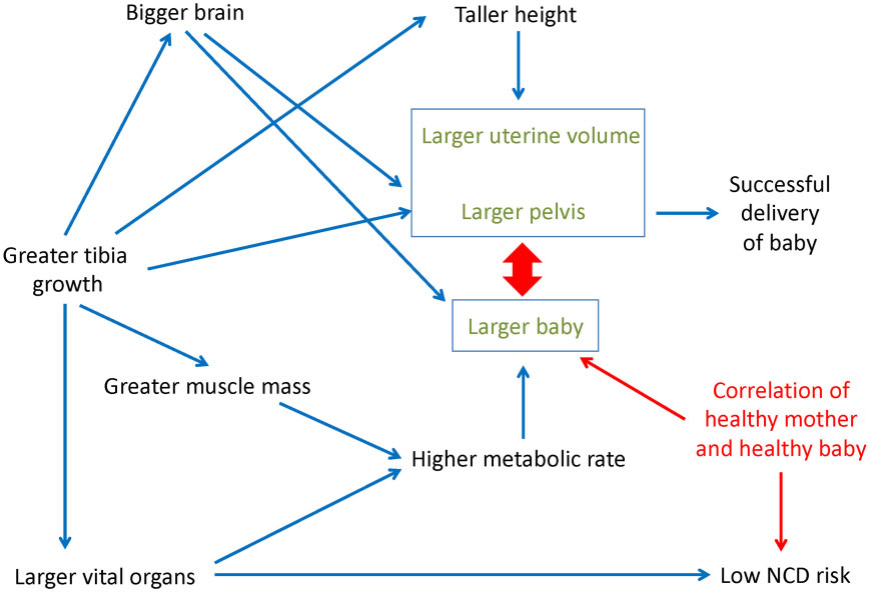
Schematic diagram of how tibia/leg length, organs and other variables might interact to influence metabolic and reproductive health and fitness for mothers and their offspring. Some of the associations shown may demonstrate greater plasticity, while others may demonstrate a stronger genetic component

In a prior analysis of this sample, we tested associations of similar growth markers—tibia length, a height-residual variable and birth weight—with MRI-measured adult pelvic dimensions and found stronger associations for tibia length and height-residual relative to birth weight [23]. Taken together, results of the prior and present analyses suggest that growing a longer tibia—and more broadly, longer legs—is associated both with a greater capacity to fund fetal growth (via increased metabolic capacity) and also a larger aperture for the fetus to exit at birth. It was already recognized that lean mass scales closely with height [45] and that the height of adult women is associated with their pelvis size [46]. However, the fact that tibia length is associated with both lean mass and pelvic dimensions suggests a degree of coordination in the development of these phenotypic traits, possibly centered on the fetal/postnatal, pre-pubertal growth period. Figure 3 is a schematic diagram of potential associations among the tibia, organs, skeletal muscle and other variables contributing to metabolic and reproductive outcomes. Some correlations may be more influenced by genes, for example, the link between head size and pelvis size [47]. Other relationships may demonstrate different degrees of plasticity. Those that are more plastic would be predicted to respond to environmental and nutritional influences in early life, and to varying degrees, within other growth periods.

A strength of our study is the use of high-quality measurement techniques to obtain body composition, brain and REE outcomes, all of which were collected by a single observer. We made an effort to control for potentially confounding variables in our study design and there were few missing data. At the same time, birth weight may have suffered from recall bias, with additional noise in the data potentially impacting our findings. Our sample size was relatively small, and, as noted previously, a cross-sectional design and the use of growth proxy measures may be considered a limitation [23]. For example, the potential for the tibia to also incorporate genetic effects cannot be discounted.

Longitudinal studies are needed to further elucidate how different growth periods shape aspects of adult phenotype, including organs, SMM and pelvis size. Although leg length–adult health associations have been similarly documented in differentially affluent populations [48], there has been some inconsistency across countries [49] and additional work is needed to understand global variation in the manifestation of these associations and their potential causes. Finally, our data do not allow us to disentangle the degree to which observed associations are influenced by plasticity, genetics, epigenetics, other factors or a combination of factors, and this is clearly a critical question for further research.

## CONCLUSIONS AND IMPLICATIONS

Despite limitations, we believe the results presented here are an important addition to the literature. These results are, to our knowledge, the first to demonstrate associations of tibia length—a sensitive component of the leg jointly indexing fetal life, infancy and early childhood—with adult organs, brain size, SMM and REE. Height residual as a marker of later growth, and birth weight, by contrast, demonstrated weaker associations. Our findings may aid in explaining previously observed associations of leg length with both adult metabolic health outcomes and offspring birth weight. They further support the importance of targeting interventions in early life [38, 50] so as to promote achievement of individuals’ genetic potential for height, organ size, lean mass and metabolic capacity more broadly. The notion that this will contribute to greater health and fitness across generations is supported jointly by our results and those previously reported, although further investigations in diverse populations are warranted.

## Supplementary Material

eoac023_Supplementary_Data

## Data Availability

Data are available upon request.
