# Supplementary material for: Implications of leg length for metabolic health and fitness
Source: Evol Med Public Health. 2022 Jul 21;10(1):316–24. doi: 10.1093/emph/eoac023 (PMC9326181; doi:10.1093/emph/eoac023)
Supplement: eoac023_Supplementary_Data [file eoac023_supplementary_data.zip › supplementary_tables_FINAL.docx]

**Supplementary Table 1.** Descriptive statistics for the sample

| Subject characteristic | *n* | Mean ± SD | Range |
| --- | --- | --- | --- |
| Age, y | 70 | 24 ± 2.4 | 20 to 28 |
| Birth weight, kg | 69 | 3.2 ± 0.5 | 2.0 to 4.5 |
| Gestational age, wks | 67 | 39.3 ± 1.5 | 34 to 42 |
| Height, cm | 70 | 161 ± 6.6 | 148 to 177 |
| Weight, kg | 70 | 57.8 ± 9.2 | 40.7 to 81.1 |
| BMI, kg/m^2^ | 70 | 22.2 ± 3.5 | 17.2 to 30.3 |
| Tibia length, cm | 70 | 36.8 ± 2.5 | 30.0 to 42.9 |
| FM, kg | 70 | 20.3 ± 6.7 | 8.3 to 40.1 |
| FFM, kg | 70 | 37.5 ± 4.3 | 28.4 to 48.8 |
| SMM, kg | 70 | 15.3 ± 2.2 | 10.8 to 20.2 |
| Heart, cm^3^ | 69 | 499 ± 88.7 | 330 to 780 |
| Liver, cm^3^ | 70 | 1139 ± 201 | 722 to 1599 |
| Kidney, cm^3^ | 70 | 277 ± 48.4 | 197 to 453 |
| Spleen, cm^3^ | 68 | 132 ± 46.0 | 72.7 to 310 |
| Brain, cm^3^ | 70 | 1041 ± 78.4 | 851 to 1210 |
| REE, kcal/day | 68 | 1337 ± 184 | 993 to 2034 |

BMI, body mass index; FM, fat mass; FFM, fat-free mass; SMM, skeletal muscle mass; REE, resting energy

expenditure

**Supplementary Table 2.** Pearson correlations of body composition variables and REE with birth weight, tibia length, height-residual, and height

|  | Birth weight (kg)  r; *p;*  95% CI | Tibia length (cm)  r; *p;*  95% CI | Height-residual (cm)  r; *p;*  95% CI | Height (cm)  r; *p;*  95% CI |
| --- | --- | --- | --- | --- |
| Weight, kg | 0.12; 0.31;  -0.12, 0.35 | 0.18; 0.14;  -0.06, 0.40 | 0.38; 0.001;  0.16, 0.56 | 0.34; 0.005;  0.11, 0.53 |
| Height, cm | 0.07; 0.55;  -0.17, 0.31 | -- | -- | -- |
| FM, kg | 0.07; 0.59;  -0.17, 0.30 | -0.005; 0.97;  -0.24, 0.23 | 0.26; 0.03;  0.03, 0.47 | 0.12; 0.33;  -0.12, 0.34 |
| FFM, kg | 0.16; 0.18;  -0.08, 0.39 | 0.41; <0.001;  0.19, 0.58 | 0.39; <0.001;  0.17, 0.57 | 0.54; <0.001;  0.35, 0.69 |
| SMM, kg | 0.08; 0.53;  -0.16, 0.31 | 0.40; <0.001;  0.18, 0.58 | 0.29; 0.01;  0.06, 0.50 | 0.49; <0.001;  0.29, 0.65 |
| Heart, cm^3^ | 0.18; 0.13;  -0.06, 0.40 | 0.43; <0.001;  0.21, 0.60 | 0.26; 0.03;  0.02, 0.47 | 0.50; <0.001;  0.30, 0.66 |
| Liver, cm^3^ | 0.08; 0.54;  -0.16, 0.31 | 0.36; 0.002;  0.14, 0.55 | 0.29; 0.02;  0.06, 0.49 | 0.46; <0.001;  0.25, 0.63 |
| Kidney, cm^3^ | 0.12; 0.34;  -0.12, 0.34 | 0.37; 0.002;  0.15, 0.56 | 0.19; 0.11;  -0.04, 0.41 | 0.42; <0.001;  0.20, 0.59 |
| Spleen, cm^3^ | 0.11; 0.37;  -0.13, 0.34 | 0.30; 0.01;  0.06, 0.50 | 0.11; 0.39;  0.11, 0.39 | 0.31; 0.009;  0.08, 0.51 |
| Brain, cm^3^ | 0.03; 0.78;  -0.20, 0.27 | 0.37; 0.001;  0.15, 0.56 | 0.17; 0.17;  -0.07, 0.39 | 0.41; <0.001;  0.19, 0.59 |
| REE, kcal/day | 0.14; 0.26;  -0.10, 0.37 | 0.36; 0.003;  0.13, 0.55 | 0.27; 0.02;  0.04, 0.48 | 0.45; <0.001;  0.24, 0.62 |

FM, fat mass; FFM, fat-free mass; SMM, skeletal muscle mass; REE, resting energy expenditure

**Supplementary Table 3.** Pearson correlations of body composition variables and resting energy expenditure with relative leg length (ratio of leg length to total height)

|  | Relative leg length  r; *p* (95% CI) |
| --- | --- |
| Weight, kg | -0.16; 0.17 (-0.38, 0.07) |
| Height, cm | 0.44; <0.001 (0.23, 0.61) |
| Fat mass, kg | -0.26; 0.03 (-0.46, -0.02) |
| Fat-free mass, kg | 0.05; 0.67 (-0.19, 0.28) |
| Skeletal muscle mass, kg | 0.13; 0.27 (-0.10, 0.36) |
| Heart, cm^3^ | 0.18; 0.13 (-0.06, 0.40) |
| Liver, cm^3^ | 0.10; 0.39 (-0.13, 0.33) |
| Kidney, cm^3^ | 0.08; 0.52 (-0.16, 0.31) |
| Spleen, cm^3^ | 0.31; 0.01 (0.07, 0.51) |
| Brain, cm^3^ | 0.21; 0.08 (-0.03, 0.42) |
| Resting energy expenditure, kcal/day | 0.14; 0.27 (-0.11, 0.36) |
