# Supplementary figures and images for: Implications of leg length for metabolic health and fitness
Source: Evol Med Public Health. 2022 Jul 21;10(1):316–24. doi: 10.1093/emph/eoac023 (PMC9326181; doi:10.1093/emph/eoac023)

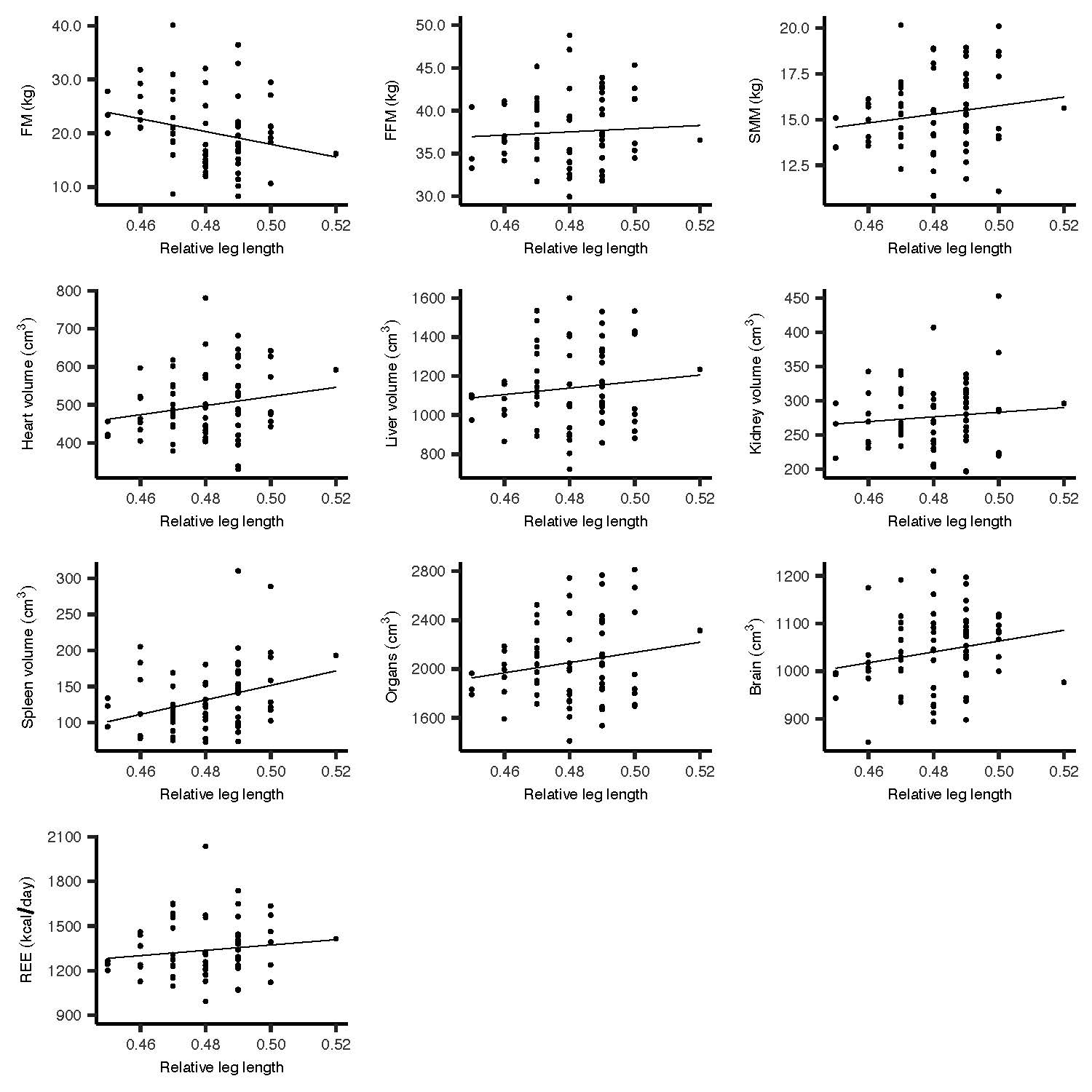

Supplement: eoac023_Supplementary_Data [file eoac023_supplementary_data.zip › SuppFigure_1.jpg]
